# A Novel Fast Servo Tool Device with Double Piezoelectric Driving

**DOI:** 10.3390/mi14010085

**Published:** 2022-12-29

**Authors:** Junfeng Liu, Tiancong Luo, Kexian Liu, Tao Lai, Yuqian Zhao, Linfeng Wang

**Affiliations:** 1 Laboratory of Science and Technology on Integrated Logistics Support, College of Intelligence Science and Technology, National University of Defense Technology, Changsha 410073, China; 2 Beijing Zhenxing Institute of Metrology and Measurement, Beijing 100074, China

**Keywords:** FTS, double piezoelectric driving (DPD), hysteresis model, microstructure processing

## Abstract

The fast tool servo (FTS) technology has unique advantages in the machining of complex surfaces such as special-shaped targets and free-form surfaces. In view of the shortcomings in the performance of the existing FTS device, this paper puts forward a novel FTS which uses two piezoelectric ceramics instead of flexure hinges to provide restoring force. Firstly, the feasibility of the double-drive principle is verified theoretically, and the corresponding mechanism is optimized accordingly. Then, the system control hysteresis model is established and identified, and the appropriate control strategy is designed. Finally, the performances of the proposed FTS device are tested, and a typical microstructure is machined based on the device and ultra-precision lathe. The results indicate that the proposed device effectively improves the performance of the FTS system, which is useful for the processing of microstructures and free-form surfaces.

## 1. Introduction

Inertial confinement fusion is an innovative technology that uses laser as the driving source to realize controlled thermonuclear fusion. It is of strategic significance to the national military, defense and economy. The machining ability of high-precision and complex-shaped special-shaped target parts is the key to this technology [1,2,3]. The application of the free-form surface makes the optical imaging system develop towards the direction of over the horizon, high resolution, large field of view, wide spectrum and dexterity [4]. It has been widely used in military investigations, missile navigation, geological remote sensing, disaster relief and risk avoidance, and has become an important field of competition among countries [5].

Traditional single-point diamond turning is difficult to ensure the manufacturing accuracy and efficiency in the processing of microstructure optical arrays and the compensation of asymmetric errors of free-form surfaces. The FTS technology with non-rotary-symmetric surface processing ability can be applied to the compensation processing of such surface error, so as to realize the direct forming of the near-infrared band mirror, which provides a high-precision and high-efficiency processing ideal [6].

In the 1980s, the Lawrence National Laboratory of micro feed technology first developed a micro-piezoelectric knife drive system in the United States. After more than 30 years of development, a series of high-performance micro-nano feed systems for FTS and a set of mature manufacturing theory and processing technology systems have been developed [7,8,9,10,11]. Liu J F et al. developed a high-frequency response and a large stroke fast knife servo device, and machined a submicron precision microstructure array on the cylindrical surface [12,13]. Xu Q H designed a kind of FTS device with DPD in the same direction and improved the performance of the device, but the structure is complex, so it is difficult to be popularized in practical machining [14].

Based on a three-axis ultra-precision lathe, Zou X C compensated the over-cut convex sphere with a curvature radius of 250 mm and a diameter of 70mm, and the surface shape accuracy of the workpiece was improved by 39.19% [15]. The Korean robotics and manufacturing technology center adopted the FTS system with a stroke of 7.5 μm and a servo bandwidth of 100 Hz to measure and compensate for the axial error of the machine tool in real time, and processed a large aspheric off-axis aluminum mirror with the diameter of 620 mm and the shape accuracy of 0.7 μm [16]; Gao W et al. measured and analyzed the contour error in the vector height direction of the surface caused by the error movement of the x-axis and the spindle, and used the developed FTS system to compensate the large sinusoidal ray surface, whose peak-valley (PV) value was reduced from 0.27 μm to 0.12 μm [17].

Various FTS systems and their applications were deeply investigated by the research above, while the FTS system reported in the public literature cannot take into account the large stroke, high response and high precision, which limits its application range and efficiency. In order to eliminate the displacement loss and improve motion accuracy, this paper designs a novel displacement guiding mechanism driven by dual piezoelectric ceramics, and realizes the servo control of FTS device with DPD. Finally, the performance improvement of the device is verified by the performance test and microstructure machining experiment.

## 2. Design and Analysis of the FTS Device with DPD

### 2.1. Operational Principle of the FTS Device with DPD

**Figure 1:**
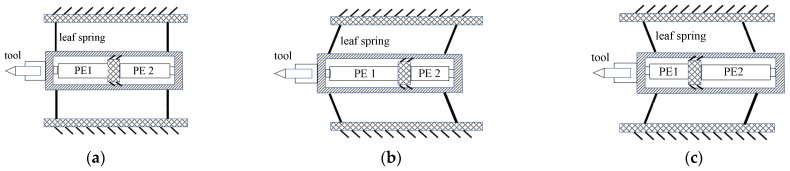
Operational principle of coaxial dual piezoelectric ceramic FTS. (**a**) middle (**b**) feed (**c**) retraction.

As shown in Figure 1, the two piezoelectric ceramics 1 and 2 extend by the same distance, so that the tool is in the middle position. If the piezoelectric ceramic PE1 extends and the PE2 shrinks by the same length at this time, the tool will complete the feed action under the driving force of PE1. On the contrary, the tool will complete the retraction action under the driving force of PE2. The reciprocating motion of the tool can be realized by controlling the opposite asynchronous telescopic movement of the above two ceramics. Since the flexure hinge is no longer required to provide the restoring force, displacement loss can be eliminated by reducing the stiffness of the flexure hinge in the tool feed direction.

**Figure 2:**
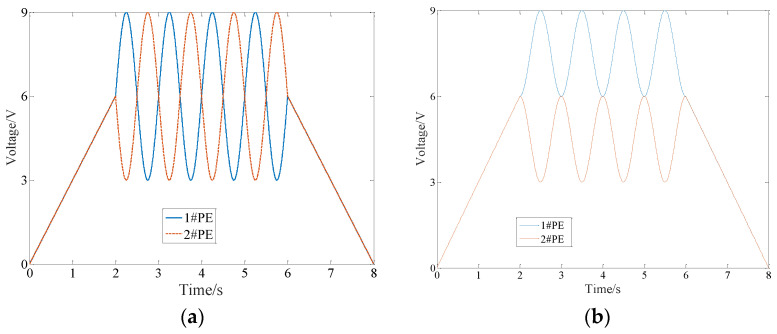
Typical signals under working state. (**a**) typical signal 1 (**b**) typical signal 2.

Therefore, the control signal of the FTS device with DPD should meet the following equation:

(1) $$f_{1}(t)+f_{2}(t)=2l$$

where *f*_1_(*t*) and *f*_2_(*t*) are the control signals of PE1 and PE2, respectively, and *l* is the pre-elongation distance of the two PEs under working state. Figure 2 shows the typical control signal, where 0 to 2 s is the system pre-tightening process, 2 to 6 s is the system movement process and 6 to 8 s is the system unloading pre-tightening process.

### 2.2. Design and Analysis of the FTS Device with DPD

According to the mechanical dynamic model of the single piezoelectric ceramic FTS device [12], the driving force of the tool feeding is provided by the piezoelectric ceramic, but the restoring force of the tool withdrawing is provided by the flexure hinge. Therefore, the mechanical model of the system in the process of tool feeding and tool retraction is actually different. The actual output displacement of the tool rest during tool retraction is related to the initial position and the structural parameters, while the initial position of the tool retraction is uncontrollable. It will lead to the asymmetry of the displacement in the two processes, increase the difficulty of control, and affect the tracking accuracy of the tool on complex surfaces.

**Figure 3:**
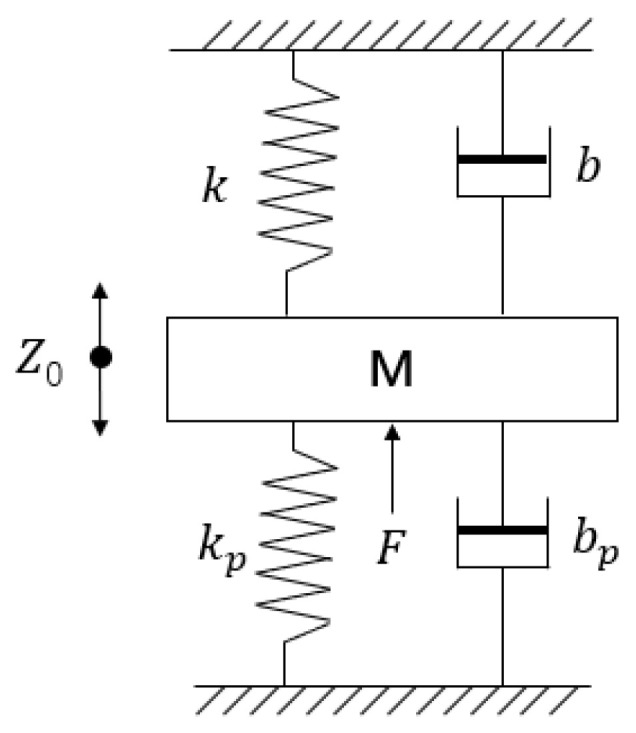
Mechanical model of DPD system.

In the micro nano feed system driven by dual piezoelectric ceramics, assuming that piezoelectric ceramics 1 and 2 can realize the ideal movement of Equation (1), the system is a mechanical model as shown in Figure 3. No matter the process of feeding or retraction, its force balance equation is the same as that of the single ceramic driving system [12]. That is, the transfer functions of the actual output displacements of the tool holder of the feeding and retraction are the same, and are only related to the structural parameters of the system, which greatly improves the accuracy and controllability of output displacement.

Where *M* is the mass of the motion module; *k* and *k_p_* are the stiffnesses of the flexure hinge and piezoelectric ceramic, respectively; *b* and *b_p_* are the dampings of the flexure hinge and piezoelectric ceramic, respectively; *Z*_0_ is the actual output displacement; *F* is the driving force.

**Figure 4:**
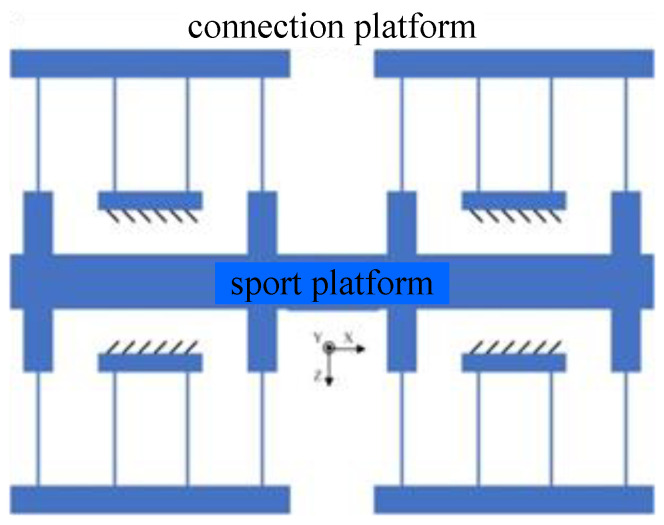
Composite double parallelogram mechanism.

The design core of the tool clamping device is the guiding mechanism with high off-axis stiffness, low feed stiffness and high motion accuracy. The low feed stiffness ensures no displacement loss in the process of transferring displacement, and the large off-axis stiffness suppresses the parasitic displacement caused by torque and impact in tool cutting. Therefore, a composite double parallelogram mechanism, as shown in Figure 4, is designed. 

The static simulation analysis of the displacement guiding mechanism is carried out to verify the rationality of the design of the displacement guiding mechanism. The material of the mechanism is 65 Mn. According to the design index of the mechanism, the value of feed stiffness is less than 5 N/μm while the value of off-axis stiffness is greater than 50 N/μm, and the working bandwidth is 200 Hz.

**Figure 5:**
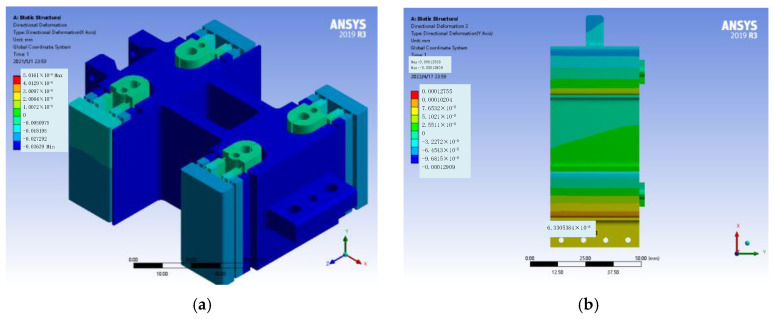

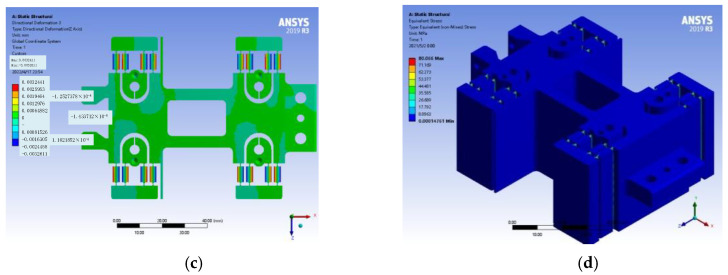
Deformation and stress diagram of the mechanism. (**a**) deformation diagram in X direction; (**b**) deformation diagram in Y direction; (**c**) deformation diagram in Z direction; (**d**) isokinetic cloud chart.

In order to analyze the feed stiffness and parasitic displacement, a force of 100 N is applied to the rear end face of the mechanism along the X direction. In addition, the mesh grid size and the element type are 10 mm and 10 node 187, respectively, and the constraints are applied on the four outer surfaces of the structure. From Figure 5, it can be seen that under the action of 100N force, the shape variables in X (feed direction), Y and Z directions are 36.39 μm, 63.31 nm and 1.434 nm, respectively. The feed stiffness is 2.748 N/μm, and the parasitic displacement in Y and Z directions is 0.174% and 0.004% of the feed displacement, respectively. The maximum stress of the mechanism occurs at the connection between the flexible beam and the platform, and its stress value is 80.066 mpa. The yield strength of 65Mn is 430 Mpa, so the safety factor is 5.37, which can meet the requirement that the value of the safety factor is greater than 3.

Similarly, by applying 100 N force along the Y and Z directions at the eight bolt holes of the mechanism, the off-axis stiffnesses of the mechanism in the Y and Z directions are 111.682 N/μm and 74.019 N/μm, respectively, and the equivalent stress safety factors are 29.92 and 31.84, respectively, which both meet the design index.

**Figure 6:**
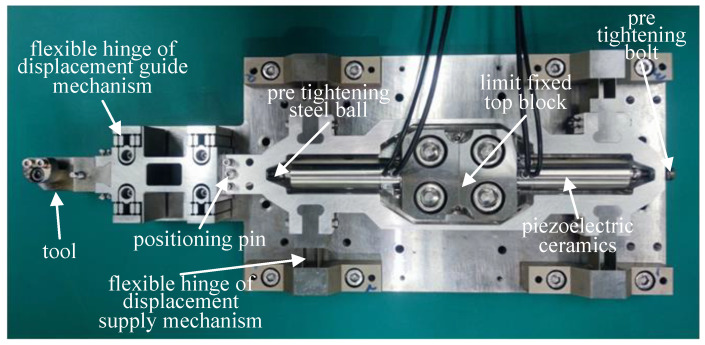
DPD system.

A DPD system is proposed based on the designed displacement guiding mechanism. As shown in Figure 6, the two piezoelectric ceramics are compressed between the motion support frame and the limit fixed top block by the pre-tightening bolts and steel balls. The reciprocating linear motion of the motion support frame in the feed direction is realized through the telescopic alternating motion of the two ceramics. At the same time, the displacement will be transmitted to the displacement guide mechanism, so as to drive the tool fixed on the adjustable tool rest to make a linear reciprocating motion, and complete the cutting motion with the cooperation of the ultra-precision machine tool. Among them, the limit fixed top block, the fixed end of the flexible hinge of the displacement supply mechanism and the fixed part of the displacement guide mechanism are fixed on the base plate by bolts, and the precise positioning relationship between each part is ensured by positioning pins.

**Figure 7:**
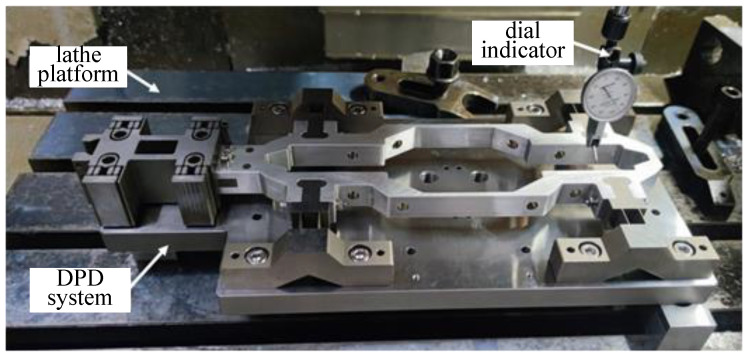
Assembly and adjustment of DPD system.

In the installation process, the displacement guide mechanism is positioned on the base plate, and assembled with the motion support frame. At the same time, the flexure hinge and the motion support frame of the displacement supply mechanism are assembled, in which the flexure hinge of the displacement supply mechanism is fixed on the base plate and positioned with a locating pin. As shown in Figure 7, the above-assembled device is put on the working platform of the ultra-precision lathe, and the machine tool and lever-dial indicator are used to level the flexible hinge and motion support frame of the displacement supply mechanism, so that its flatness is less than 20 μm. The flexible hinge of the displacement supply mechanism and the moving support frame are locked by bolts. The above assembly and adjustment process ensures the overall flatness and adaptability of the DPD system, and avoids damage to the electric ceramics due to eccentric load.

**Figure 8:**
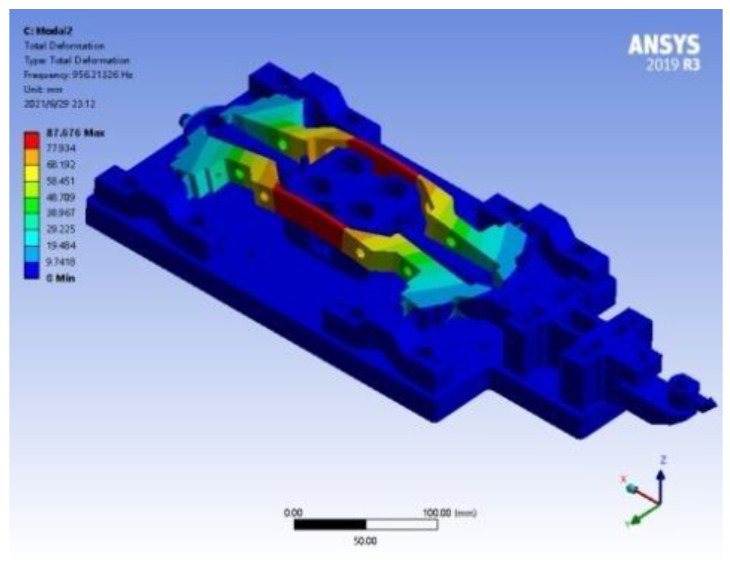
The first natural frequency and vibration mode of the mechanical of DPD.

According to the design index requirements, the DPD system needs to have a working bandwidth of 200 Hz, so it is necessary to conduct a modal analysis of its mechanical system to ensure that the first natural frequency of the system is greater than its working bandwidth. Figure 8 shows the ANSYS simulation results. It can be seen that the first-order natural frequency of the mechanical structure of the system is 956.21326 Hz, and the vibration mode is the deformation in the Z direction of the middle part of the moving support frame, which meets the design index.

## 3. Servo Control of the DPD System

For the DPD system, its servo control is faced with many difficulties, such as nonlinear effect, amplitude attenuation, phase delay, and cooperative motion. Therefore, a robust control strategy is proposed to deal with these problems. 

**Figure 9:**
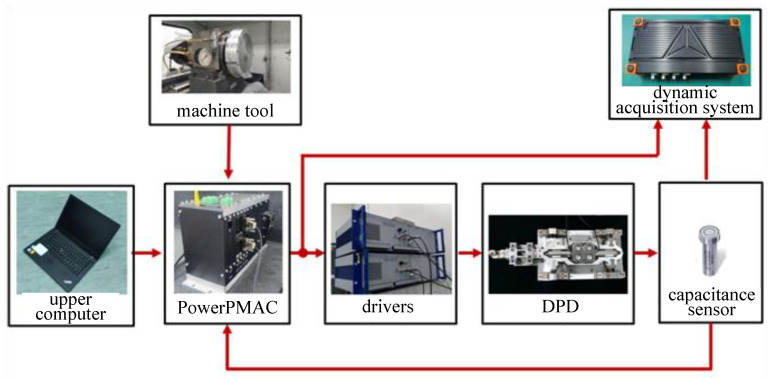
The schematic diagram of DPD control system.

The schematic diagram of DPD control system is shown in Figure 9, and its working principle is as follows. The processing program compiled by the upper computer is downloaded to PowerPMAC. The shaft card Acc24E3 of PowerPMAC receives the encoder information of the spindle speed and X-axis displacement of the ultra-precision machine tool, and the analog quantity board Acc59E3 receives the analog quantity information of the capacitance sensor PI-D-510.21. The received information is processed by the designed servo control algorithm to generate a voltage output signal, which is output by the analog quantity board Acc59E3. The voltage signal is amplified by the driver PI-E480 and input to the DPD system, and controls the system to move according to the setting to cut the workpiece. The dynamic acquisition system DEWESOFT collects and stores the voltage signal output by the analog quantity board card and the displacement information during the operation of the DPD system through the shaft card Acc24E3 and the capacitance sensor, respectively. At the same time, the capacitance sensor also outputs the displacement information to the PowerPMAC, so that all the above parameters can be collected simultaneously in the dynamic acquisition system for subsequent analysis, especially synchronous performance analysis.

**Figure 10:**
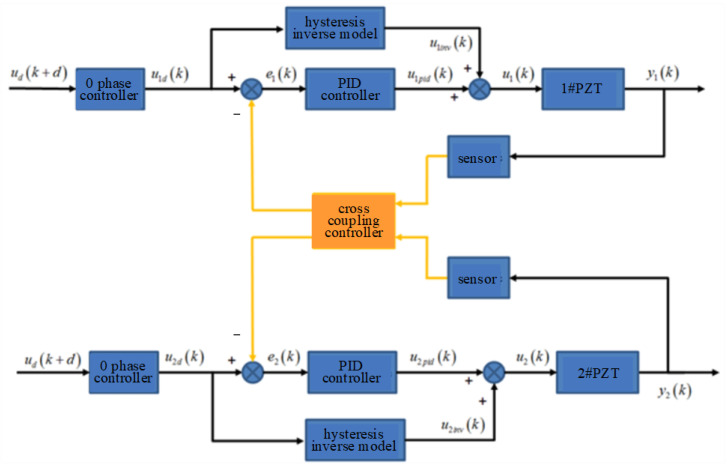
The integrated control algorithm of DPD system.

Aiming at the servo control problem of the DPD system, an integrated control algorithm is proposed as shown in Figure 10. Firstly, a feedforward control method based on the modified Prandtl Ishlinskii hysteresis inverse model is used to eliminate the influence of hysteresis nonlinearity of piezoelectric ceramics. On this basis, the PID controller is added to eliminate the influence of the modeling error of hysteresis inverse model and other disturbance errors, so as to further improve the tracking accuracy of the DPD system. At the same time, the zero phase controller is added to overcome phase delay and amplitude attenuation. Finally, a cross-coupling controller is added to the above algorithm to solve the cooperative motion required by the DPD system.

### 3.1. Algorithm Design for Compensating Nonlinearity of Piezoelectric Ceramics

The previous research work of our team shows that the Prandtl Ishlinskii model is more suitable for describing the rate of independent hysteresis phenomenon of piezoelectric ceramics [18]. Therefore, this paper designs a PID controller based on the modified Prandtl Ishlinskii (PI) inverse model, and verifies its effectiveness in compensating the nonlinearity of piezoelectric ceramics.

**Figure 11:**
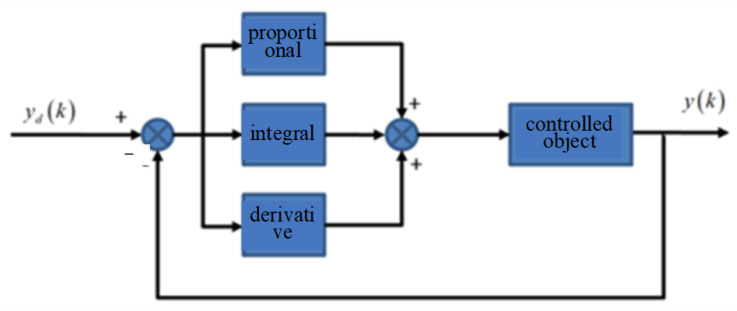
The PID controller.

The PID controller is the most widely used control method because of its simple structure, good stability, reliability, easy adjustment and other advantages. It realizes control by adjusting the proportional link *k_p_*, integral link *k_i_* and derivative link *k_d_*. Its schematic diagram is shown in Figure 11, and the discretization expression is as follows [19]:

(2) $$\left\{ \begin{array}{l} u(k)=Kp\cdot e(k)+K_{I}\cdot \sum_{i=1}^{k}e(i)+K_{D}\cdot [e(k)-e(k-1)]\\ e(k)=y_{d}\left(k\right)-y\left(k\right) \end{array}\right.$$

where *u*(*k*) is the input signal of piezoelectric ceramics, *y_d_*(*k*) and *y*(*k*) are the expected and actual output positions of piezoelectric ceramics, respectively. 

The PI model has an analytical inverse model and its structure is consistent with that of the PI model, but its threshold value and density function are different, which reduces the difficulty of solving the hysteresis inverse model. The expression of the inverse model is:

(3) $$\begin{array}{ll} u(k) & =Y^{-1}\left[y(k)\right]\\ & =\sum_{i=0}^{N-1}w_{i}^{\prime }\max\{y(k)-r_{i}^{\prime },\min\{y(k),u(k-1)\}\} \end{array}$$

The initial value in the formula is calculated as follows:

(4) $$\left\{ \begin{array}{l} w_{i}^{\prime }=\frac{-w_{i}}{\left(\sum_{j=1}^{i}w_{j}\right)\left(\sum_{j=1}^{i-1}w_{j}\right)}\\ r_{i}^{\prime }=\sum_{j=1}^{i}w_{j}\left(r_{i}-r_{j}\right)\\ w_{1}^{\prime }=\frac{1}{w_{1}}\\ u_{i}\left(0\right)=\sum_{j=1}^{i}w_{j}y_{i}\left(0\right)+\sum_{j=i+1}^{n}w_{j}y_{j}\left(0\right) \end{array}\right.$$

where *i* = 1, 2, 3, …, *n*.

**Figure 12:**
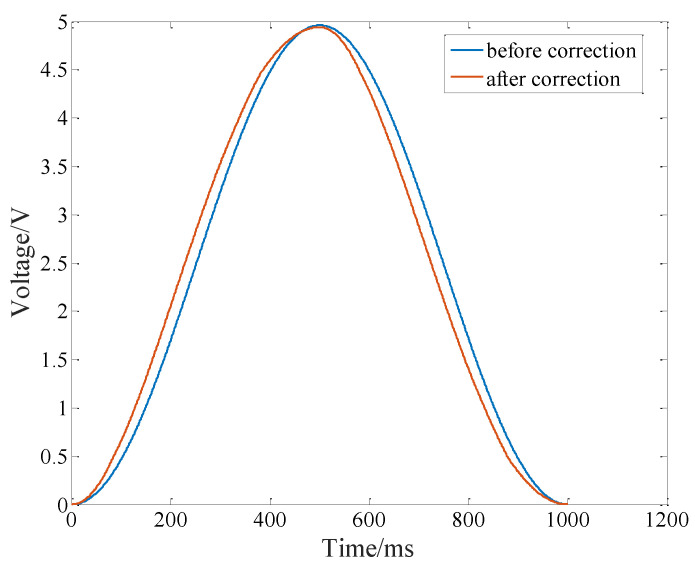
Input signals before and after correction of PI inverse model.

The input signals before and after the correction of the inverse model are obtained according to the model parameters [18], as shown in Figure 12.

**Figure 13:**
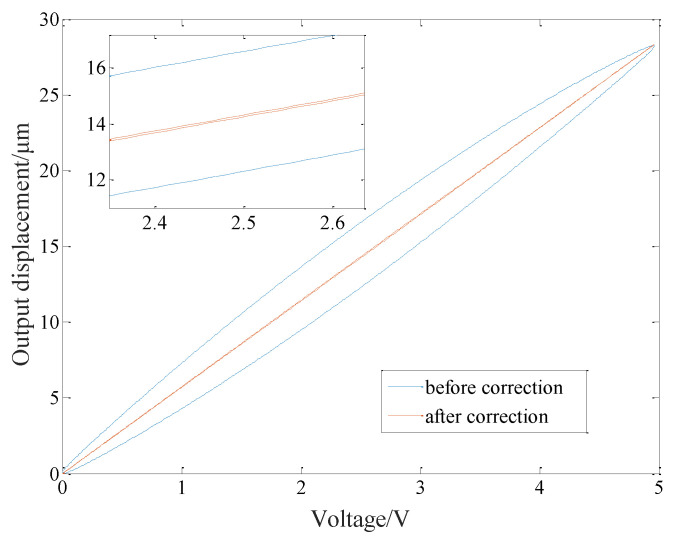
Hysteresis cycle before and after correction of PI inverse model.

The PI inverse model is used to correct the hysteresis nonlinearity of ceramics, and the results is shown in Figure 13. The maximum hysteresis error is 4.283 μm down to 0.077 μm, and the hysteresis phenomenon is reduced by 98.199%, which proves the effectiveness of the proposed hysteresis inverse model.

**Figure 14:**
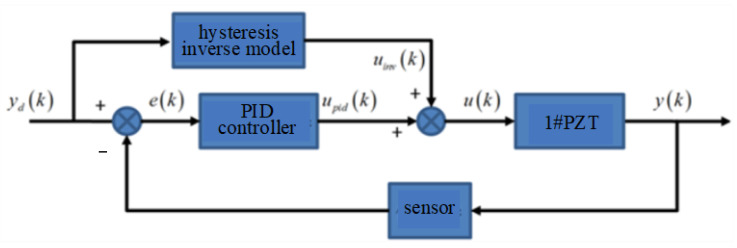
PID control flow chart based on PI inverse model.

Due to the strong nonlinearity of piezoelectric ceramics, the feedforward control using only the PI inverse model cannot compensate for the hysteresis modeling error and the errors caused by temperature, disturbance, environment and other factors. A PID control algorithm based on PI inverse model is proposed as shown in Figure 14. The PI inverse model compensates for most of the nonlinear errors of piezoelectric ceramics, and the PID controller compensates for the remaining errors.

In the figure, *u*(*k*) is compose of *u_inv_*(*k*) and *u_pid_*(*k*) and its expression is:

(5) $$\left\{ \begin{array}{l} u_{inv}\left(k\right)=\sum_{i=0}^{N-1}w_{i}^{\prime }\max\{y_{d}(k)-r_{i}^{\prime },\min\{y_{d}(k),u_{inv}(k-1)\}\}\\ u_{pid}(k)=K_{p}\cdot e(k)+K_{I}\cdot \sum_{i=1}^{k}e(i)+K_{D}\cdot [e(k)-e(k-1)]\\ e(k)=y_{d}\left(k\right)-y\left(k\right)\\ u\left(k\right)=u_{inv}\left(k\right)+u_{pid}(k) \end{array}\right.$$

where *u_inv_*(*k*) and *u_pid_*(*k*) are the output signals by *y_d_*(*k*) through the hysteresis inverse model and PID controller, respectively.

**Figure 15:**
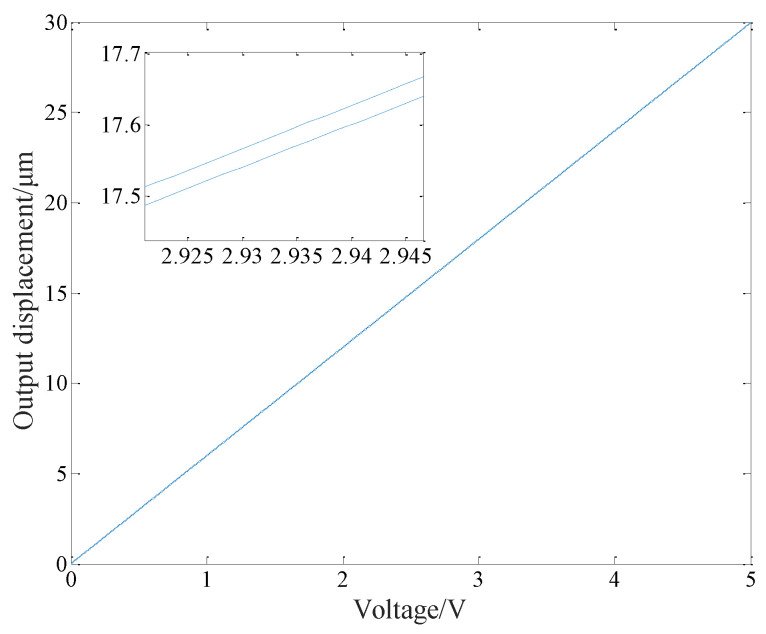
Hysteresis curve under PID control based on PI inverse model.

Write the control algorithm shown in Equation (5) into the PowerPMAC motion controller, input a 1 Hz sine signal with an amplitude of 5 V, and the sensor collects the output of piezoelectric ceramics. The result is shown in Figure 15. It can be seen that PID control based on PI inverse model has a good control effect under static signals. Its maximum hysteresis error is 0.029 μm and the relative hysteresis error is 0.1%. With this algorithm, the hysteresis nonlinearity of piezoelectric ceramics is basically eliminated. 

### 3.2. Algorithm Design for Compensating Amplitude Attenuation and Phase Delay of Piezoelectric Ceramics

Aiming at the problem of amplitude attenuation and phase delay of piezoelectric ceramics, a zero-phase controller is proposed to overcome the influence of this problem on servo control accuracy. 

**Figure 16:**
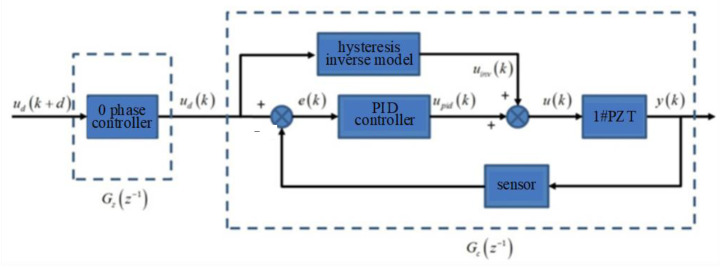
Zero phase control flow chart based on PI inverse model.

Because the DPD system is based on the synchronous motion of two piezoelectric ceramics, the control effect of each piezoelectric ceramic should be described so that the synchronization of two piezoelectric ceramics can be analyzed later. The flow chart of the zero-phase controller of a single piezoelectric ceramic is shown in Figure 16.

The sweep frequency and identification experiments are carried out on piezoelectric ceramics No.1 and No.2 [18], and the control effect is shown in Table 1. It can be seen that the zero-phase controller improves the tracking accuracy of the DPD system and compensates for the errors caused by the amplitude attenuation and phase delay of piezoelectric ceramics.

### 3.3. Algorithm Design for Restraining Synchronization Error of DPD System

**Figure 17:**
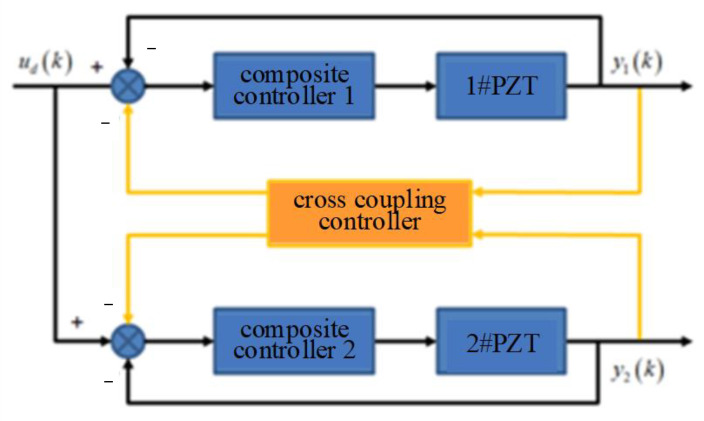
The diagram of cross-coupling controller.

The synchronization error of the DPD system will lead to system error and instability, and may damage the mechanical structure of the system. The cross-coupling controller is used to coordinate and compensate for the output error of the two piezoelectric ceramics, so as to suppress the synchronization error of the two piezoelectric ceramics. The flow diagram is shown in Figure 17.

The cross-coupling controller in the figure can be expressed as:

(6) $$C_{h}=\left[\begin{array}{l} y_{1}\\ y_{2} \end{array}\right]+{\left[\begin{array}{l} ccc_{1}\\ ccc_{2} \end{array}\right]}^{T}\left[\begin{array}{l} \varepsilon_{1}\\ \varepsilon_{2} \end{array}\right]$$

where the cross-coupling coefficients **C** = [*ccc*_1_ *ccc*_2_], **Y** = [*y*_1_ *y*_2_]^T^; *y*_1_ and *y*_2_ are the actual outputs of two piezoelectric ceramics, and the synchronization error is defined as:

(7) $$\left[\begin{array}{l} \varepsilon_{1}\\ \varepsilon_{2} \end{array}\right]=\left[\begin{array}{cc} 1 & -1\\ -1 & 1 \end{array}\right]\left[\begin{array}{l} y_{1}\\ y_{2} \end{array}\right]$$

Equation (6) can be obtained by sorting:

(8) $$C_{h}=\left(I+CT\right)Y$$

**Figure 18:**
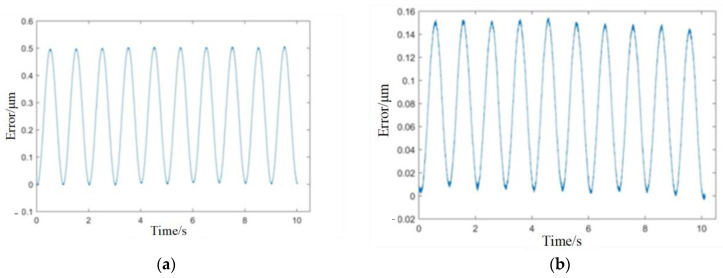
Effect of cross-coupling controller. (**a**) without cross-coupling controller; (**b**) with cross-coupling controller.

The cross-coupling controller is designed according to Equation (8). In order to simulate the error caused by the different loading conditions and disturbances of the two piezoelectric ceramics, a sinusoidal voltage with an amplitude of 0.1 V and frequency of 1 Hz is input to No.1 piezoelectric ceramics as a disturbance, and no signal is input to No.2 piezoelectric ceramics. Figure 18 shows the influence of the cross-coupling controller on the error between two piezoelectric ceramics, the maximum error switches from 0.5 μm to 0.155 μm. The error is reduced by 69%, and the effect is significant.

**Figure 19:**
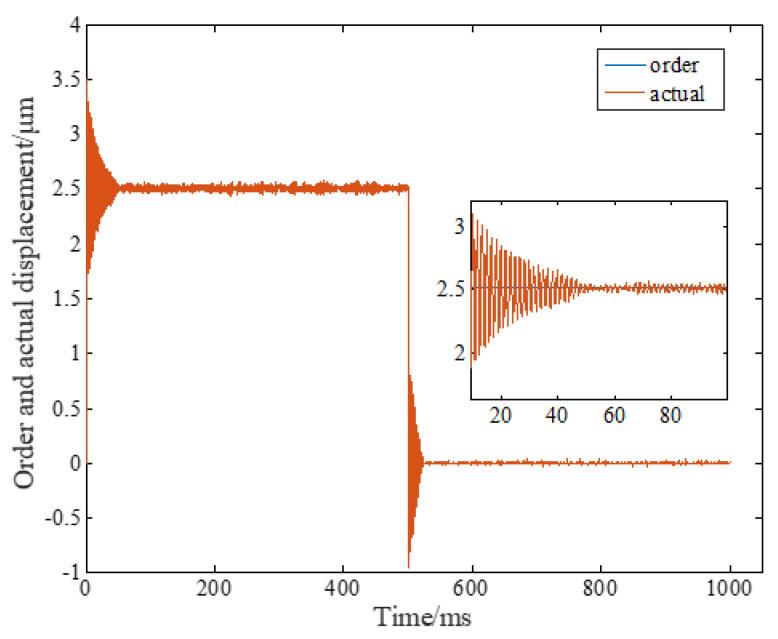
Effect of cross-coupling controller.

It is worth noting that the cross-coupling controller will change the stability of the original system, so it is necessary to select an appropriate cross-coupling coefficient **C**, otherwise it will cause system vibration or instability. Figure 19 shows that the large cross-coupling coefficient causes the step response of piezoelectric ceramics to vibrate, which seriously affects the control accuracy and even damages the equipment. Therefore, additional attention should be paid to the design of cross-coupling coefficient.

## 4. Performance Test and Processing Experiment of DPD System

### 4.1. Stiffness Test of the Hinge

Hinge stiffness is the precondition to ensure the behaviors of the DPD system, and it is measured to verify the rationality of the mechanical design of the system. The stiffness measurement formula is *K* = *F*/*S*, where *K* is the stiffness; *F* is the applied load and *S* is the displacement in the direction of the applied load.

**Figure 20:**
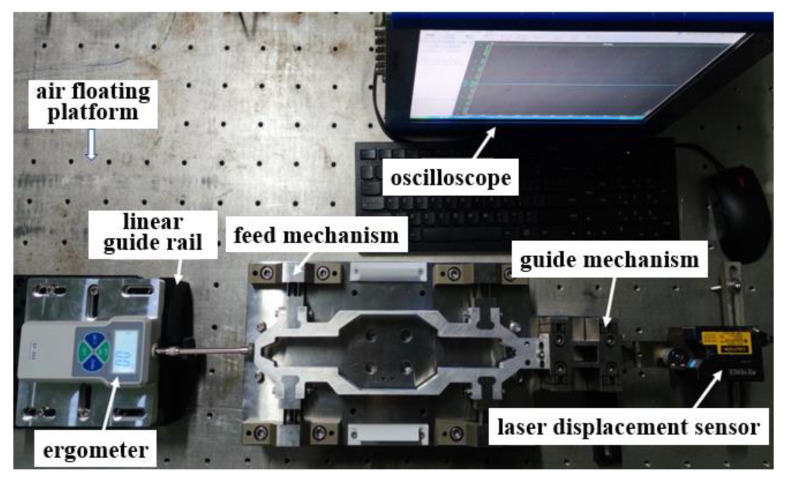
Stiffness measurement processing.

Figure 20 shows the stiffness measurement processing. The linear guide rail is used to apply the load; meanwhile, the ergometer SF-305 made in China and oscilloscope TDS7054 made by Tektronix in America collect and display the load value, respectively, and the laser-displacement sensor KEYENCE LJ-X8000 made in Germany is used to measure the displacement in the load direction. Repeat the above process in the feed and off-axis directions to obtain the feed stiffness and off-axis stiffness of the hinge. The applied load in both directions starts from 0 N, and records the measurement results every 10 N in the feed direction until 100 N. In the off-axis direction, the measurement results are recorded every 100 N in the feed direction until 500 N. The stiffness values under different loads can be obtained according to the changing displacements. Taking the average value, it can be known that the feed stiffness and off-axis stiffness of the hinge are about 2.24 N/μm and 88.43 N/μm, respectively, which is in good agreement with the simulation results and meets the design indicator.

### 4.2. Cooperative Motion Experiment of DPD System

The capacitance sensor is used to measure the displacement of the motion support frame with the cooperative motion of dual piezoelectric ceramics, and the motion synchronization of the DPD system is analyzed. According to the proposed composite control algorithm, the two piezoelectric ceramics are servo controlled, and then, the PowerPMAC motion controller is used to synchronously input 1 to 200 Hz sweep signal. The amplitude change of No.1 piezoelectric ceramics is 3 V to 5.5 V, and that of No.2 piezoelectric ceramics is 0.5 V to 3 V.

**Figure 21:**
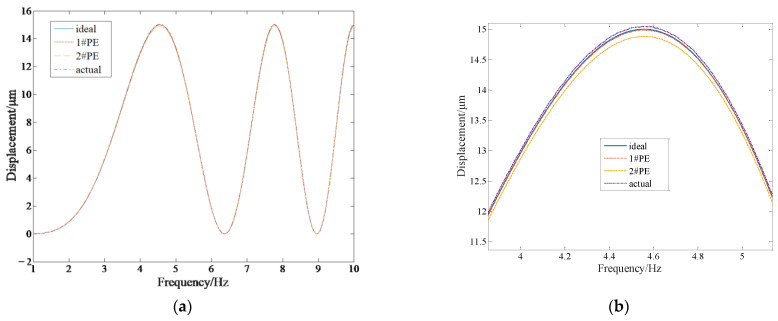

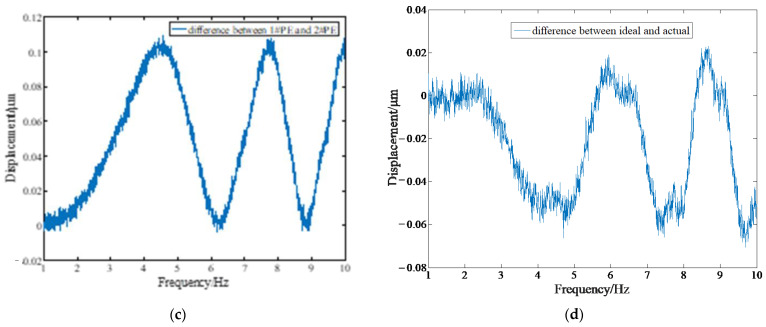
Synchronization error of DPD system from 1 Hz to 10 Hz. (**a**) output curve from 1Hz to 10 Hz; (**b**) partial enlarged view; (**c**) output difference between 1#PZT and 2#PZT; (**d**) output difference between ideal and actual.

Figure 21 shows the synchronization error of the DPD system from 1 Hz to 10 Hz. The maximum difference between the output of No.1 piezoelectric ceramic and that of No.2 piezoelectric ceramic is 0.109 μm; meanwhile, the maximum difference between the ideal output and the actual output of the motion support frame is 0.071 μm. It is known that the motion error of the motion support frame is less than the synchronous error of two piezoelectric ceramics.

The output difference between No. 1 and No. 2 piezoelectric ceramics, with an output displacement of 15 μm in different frequency bands, is shown in Table 2. When the frequency is within 200 Hz, the maximum error of motion support frame is 0.764 μm, and the relative maximum error is 5.093%. When the frequency is within 100 Hz, the maximum error of the motion support frame is 0.348 μm, and the relative maximum error is 2.32%. When the frequency is within 50 Hz, the maximum error of the motion support frame is 0.195 μm, and the relative maximum error is 1.3%. When the frequency is within 10 Hz, the maximum error of the motion support frame is 0.071 μm, and the relative maximum error is 0.473%. It can be seen that the proposed integrated control algorithm has achieved a good synchronization control effect.

### 4.3. Linearity Measurement of the DPD System

**Figure 22:**
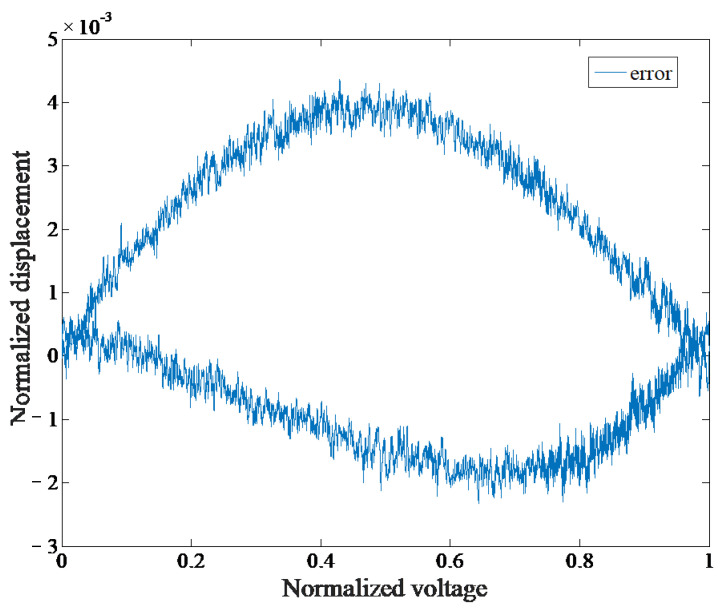
The linearity measurement curve of DPD system.

To measure the linearity of the DPD system, a triangle wave signal with a 2.5 V input amplitude and 2 s period is inputted. The output displacement of the end of the motion support frame is collected, and the input and output data are normalized. The test results are shown in Figure 22. The maximum linearity error is 0.436%, which indicates that the DPD system has good linearity.

### 4.4. Processing Experiment

In order to verify the processing performance of the DPD system in variable frequency feeds, the two-dimensional sine wave surface is selected for processing, because the surface is non-rotationally symmetric, and the feed frequency of the DPD system changes when the spindle speed is unchanged. The formula of the two-dimensional sine wave surface is:

(9) $$z(r,\theta )=A_{1}\sin(\frac{2\pi r\cos\theta }{\lambda_{1}})+A_{2}\cos(\frac{2\pi r\sin\theta }{\lambda_{2}})+\left(A_{1}+A_{2}\right)$$

where *z* is the feed rate of the DPD system; *A*_1_ and *A*_2_ are the amplitudes of sine waves in *X* and *Y* directions, respectively; *λ*_1_ and *λ*_2_ are the wavelength of the sine wave in *X* and *Y* directions, respectively. 

**Figure 23:**
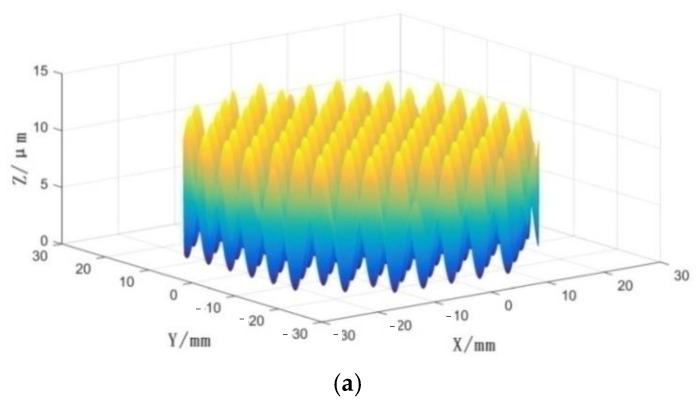

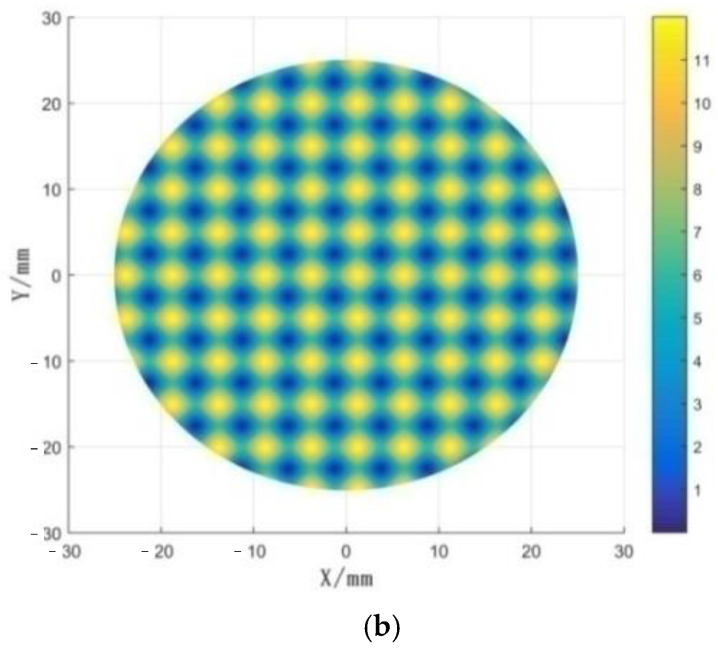
Simulation diagram of the surface. (**a**) 3D topography simulation diagram; (**b**) projection on XY plane.

Figure 23 shows the simulation diagram of the surface when the values of *A*_1_, *A*_2_, *λ*_1_ and *λ*_2_ are 3 μm, 3 μm, 5 mm and 5 mm, respectively.

The processing experiment was carried out in combination with the Nanoform 350 ultra-precision lathe, and the processing parameters are shown in Table 3.

**Figure 24:**
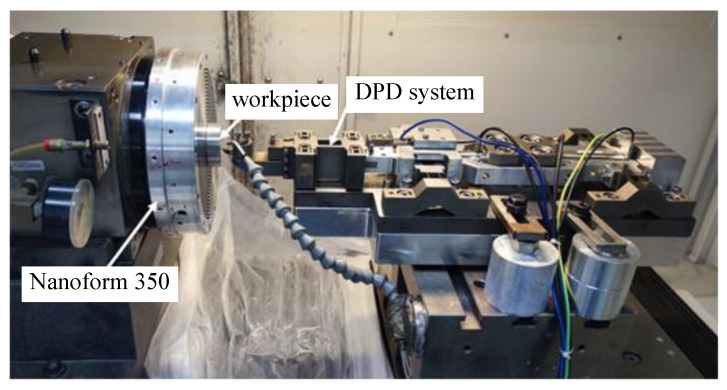
The processing site.

**Figure 25:**
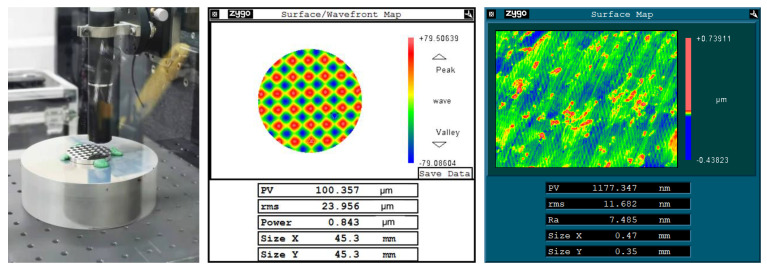
Machined workpiece and its accuracy.

The processing site is shown in Figure 24, and the processed workpiece and its surface shape and precision are shown in Figure 25. The measured surface shape is similar to the simulated surface shape, and the roughness Ra of the machined surface is 7.49 nm, which verifies the machining performance of the system in variable frequency feed. It must be said that the red spots in the figure are impurities in the workpiece material, and the surface quality can be further improved if aluminum with better material is used.

## 5. Discussion

A novel DPD system is designed and manufactured and the corresponding high-precision control algorithm is proposed, and the motion performance and processing capability of the system are verified by experiment. From the results the following conclusions are drawn:

(a) The designed composite double parallelogram flexure hinge can meet the design principle of the DPD system, and its static and dynamic behaviors are validated by simulation.
(b) The proposed integrated control algorithm is able to solve the difficulties of the DPD system, such as nonlinear effect, amplitude attenuation, phase delay and cooperative motion.
(c) The motion performance and processing experiment is carried out, and the results indicate that the DPD system can realize high-precision cooperative motion and high linearity, and has the ability to process high-precision complex microstructures.

## Figures and Tables

**Table 1:** The control effect of zero-phase controller.

	Identifier	1~5 Hz	1~20 Hz	1~200 Hz
tracking accuracy	1#PZT	0.47%	0.48%	0.83%
	2#PZT	0.77%	077%	0.96%

**Table 2:** The control effect of zero phase controller.

Frequency Range	1~200 Hz	1~100 Hz	1~50 Hz	1~10 Hz
maximum movement error of support frame/μm	0.764	0.348	0.195	0.071

**Table 3:** Processing parameters.

Material	Spindle Speed	Feed Speed	Tool Radius
AL6061	500 r/min	1 μm/min	0.5 mm

## Data Availability

Not applicable.
